# Editorial: Ecology, Metabolism and Evolution of Archaea-Perspectives From Proceedings of the International Workshop on Geo-Omics of Archaea

**DOI:** 10.3389/fmicb.2021.827229

**Published:** 2022-01-21

**Authors:** Brian P. Hedlund, Chuanlun Zhang, Fengping Wang, Christian Rinke, William F. Martin

**Affiliations:** ^1^School of Life Sciences, University of Nevada, Las Vegas, NV, United States; ^2^Nevada Institute of Personalized Medicine, University of Nevada, Las Vegas, NV, United States; ^3^Shenzhen Key Laboratory of Marine Archaea Geo-Omics, Southern University of Science and Technology, Shenzhen, China; ^4^Southern Marine Science and Engineering Guangdong Laboratory (Guangzhou), Guangzhou, China; ^5^State Key Laboratory of Microbial Metabolism, School of Life Sciences and Biotechnology, Shanghai Jiao Tong University, Shanghai, China; ^6^School of Oceanography, Shanghai JiaoTong University, Shanghai, China; ^7^Australian Centre for Ecogenomics, School of Chemistry and Molecular Biosciences, The University of Queensland, St Lucia, QLD, Australia; ^8^Institute for Molecular Evolution, University of Dusseldorf Medical School, Düsseldorf, Germany

**Keywords:** archaea, genomics, ecology, ammonia-oxidizing archaea, metagenomics, marine microbiology, extremophiles, methanogens

## Introduction

The Archaea is the most recently discovered and the least understood of life's domains. Archaea include several of the most extreme of extremophiles (e.g., Bolhuis et al., 2004; Dopson et al., 2004; Takai et al., 2008; Zeng et al., 2009; Glynn, 2017): they possess unusual physiologies (Schäfer et al., 1999; Thauer et al., 2008; Stahl and de la Torre, 2012; Evans et al., 2019; Tahon et al., 2021); perform key roles in elemental cycles in a variety of extreme environments (Baker and Banfield, 2003; de la Torre et al., 2008; Dodsworth et al., 2011; He et al., 2016; Mayumi et al., 2016; Colman et al., 2018; Hua et al., 2019); inhabit deep crust ecosystems and deep-sea hydrothermal vents (e.g., Stevens and McKinley, 1995; Ver Eecke et al., 2012), and their most ancient CO_2_ fixation pathway can be catalyzed *in vitro* by a piece of metal (Martin, 2020; Preiner et al., 2020). They have also left isotopic evidence indicating their prevalence among the most ancient microbial communities (Ueno et al., 2006; Wei et al., 2011; Schopf et al., 2018; Cavalazzi et al., 2021).

Despite fascination for their ability to conquer extremes, broader appreciation of the importance of archaea as ubiquitous members of microbial communities in “non-extreme” habitats continues to evolve in parallel with advances in environmental genomics, taxonomically resolved microbial activity studies, and laboratory cultivation studies (Wang et al., 2021a). The emerging view is that archaea are prominent members of all terrestrial and marine communities (DeLong) and play central roles in global carbon and nitrogen cycles in the ocean and on land (Hatzenpichler, 2012; Spang et al., 2017; Yu et al., 2018; Evans et al., 2019; Qin et al., 2020; Zhang et al., 2020). Recent environmental genomics on uncultured asgard archaea (Eme et al., 2017; Spang et al., 2017; van der Gulik et al., 2017; Zhou et al., 2018; Liu et al., 2021; Xie et al., 2021) and the isolation of co-cultures of the microscopically characterized obligate symbiotic archeaon *Candidatus* Prometheoarchaeum syntrophicum (Imachi et al., 2020) has led to exciting insights into the role of the Archaea in eukaryogenesis. However, most archaea remain uncultivated (Zhang et al., 2015; Spang et al., 2017; Rinke et al., 2021) and therefore the world of the Archaea remains an exciting frontier in biology.

**Figure 1:**
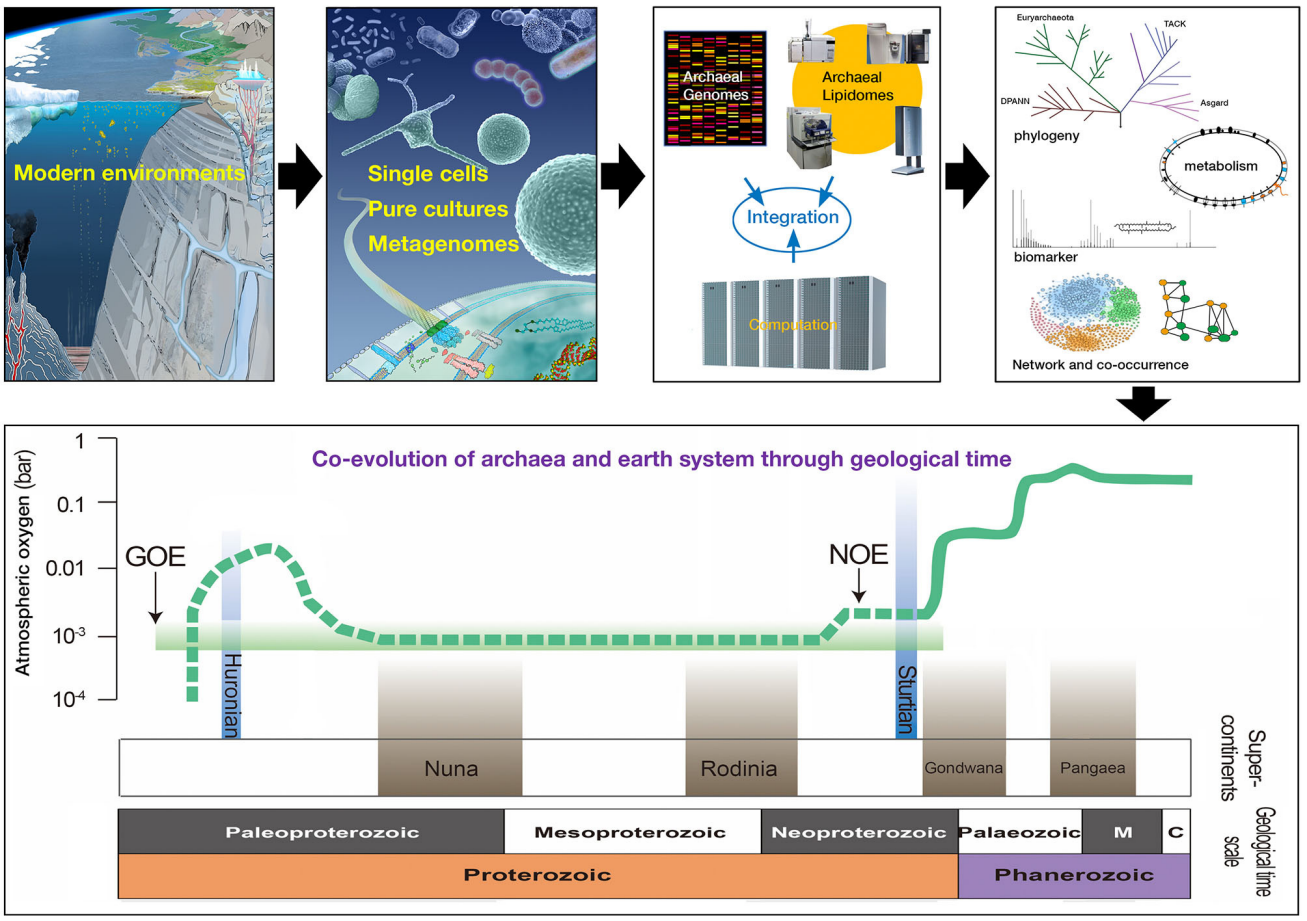
Conceptual framework to study archaeal biology. Archaea can be studied in a variety of environments in part through cultivation, environmental genomics, and lipidomics. Diverse data streams can be integrated computationally and intellectually to address questions about archaeal metabolism, evolution, and ecology. Archaeal biomarkers can be retrieved from modern and ancient systems to reconstruct the co-evolution of archaea and earth system within a geological framework.

To facilitate global efforts in addressing fundamental questions related to the biology of archaea, an international consortium of experts organized the International Workshop on Geo-Omics of Archaea (IWGOA), with the overarching themes of Ecology/Biogeochemistry, Metabolism, and Evolution. The IWGOA was held in Shenzhen, China, from October 25th to 27th, 2019. The meeting was attended by more than 200 attendees from China, Japan, USA, Australia, Germany, and France. Some of the most exciting oral and poster presentations made at the IWGOA are celebrated in this Research Topic Figure 1. The 21 manuscripts herein span different aspects of archaeal biology in both extreme and “non-extreme” environments in both marine and terrestrial settings and use a variety of approaches—community ecology, environmental lipidomics and genomics, organismal biology, and nucleic acid biochemistry—embodying diverse research thrusts that makes archaeal biology so exciting. At the same time, the manuscripts include over 100 authors from Asia, North America, and Europe, realizing our goal to engage a global audience in the biology of archaea.

## Ecology of Archaea in “Non-extreme” Marine Environments

Several papers in the Research Topic explore the ecology of archaea in marine environments. These studies are framed by a perspective article by Ed DeLong on planktonic marine archaea (DeLong). The perspective chronicles the exciting discovery in the 1990s by DeLong and contemporaries that planktonic marine archaea are not methanogens within anoxic niches in particulate organic matter, as originally hypothesized, but rather physiologically and ecologically distinct organisms, with distinct roles including chemolithoautotrophic ammonia oxidation. The perspective then carries the torch to present and future generations by highlighting the need to better understand the flood of metagenome-assembled genomes (MAGs), advance cultivation efforts, and further explore the importance of viruses infecting these organisms. A minireview follows this lead but focuses instead on archaea in estuaries, especially ammonia-oxidizing archaea (AOA) and *Bathyarchaeota* (Zou et al.). The review pegs *Nitrosopumilales* and *Nitrosospharales* as the dominant AOA in estuarine waters and sediments and identifies specific estuarine sub-lineages of AOA and *Bathyarchaeota* based on a meta-analysis of 16S rRNA gene surveys. Emerging evidence for the importance of *Bathyarchaeota* in both C1 metabolism and the consortial degradation of complex organic carbon is also reviewed.

Research papers on marine archaea included three studies on factors affecting the distribution and diversity of archaea in marine environments. One paper examined 16S rRNA genes and transcripts in samples from the epipelagic and deep chlorophyll maximum layer that were mixed to simulate upwelling by western Pacific eddies (Dai et al.). Although less abundant than AOA, heterotrophic Marine Group IIb (MGIIb) *Euryarchaeota* were enriched in response to upwelling. Two other studies focused on sediment cores collected from the Pearl River Estuary in southeastern China. The first established strong vertical stratification of dissolved organic matter (DOM) consistent with increases in recalcitrant DOM with depth, and corresponding stratification of microbial communities (Wang et al., 2021b). Lignin and carboxyl-rich alicyclic molecules were strongly correlated with *Bathyarchaeia* in deeper sediments; correspondingly, *Bathyarchaeia* MAGs were enriched with genes predicted to encode enzymes for the reductive dearomatization and carboxylation of aromatic compounds. Lai and colleagues examined vertical patterns in bathyal sediments (water depth: 2,125 m) associated with the Pearl River Submarine Canyon and associated increased terrestrial input during Pleistocene glacial maxima with increases in the terrestrial Soil Crenarchaeotic Group (SCG; *Nitrososphaerales*) along with heterotrophic *Bathyarchaeia* and *Thermoprofundales*, implying a role for terrestrial input to the deep benthos in structuring archaeal communities on millennial scales (Lai et al.).

Two studies focused on roles of archaea in C1 metabolism in marine settings. The first investigated the effects of organic phosphate addition to coastal marine sediments with or without the seagrass *Zostera marina* on methylotrophic methanogenesis (Zheng et al.). Methanogenesis was stimulated by organophosphates in both enrichments and pure cultures, but responses were both taxonomically and spatially distinct. Another study used DNA and RNA to probe community structure and activity associated with sulfate-dependent anaerobic oxidation of methane (AOM) in three cold seeps in the northern South China Sea (Zhang et al., 2020). 16S rRNA amplicons from DNA and RNA were distinct and showed three different stages of community development concordant with the development of communities of *Calyptogena* clams.

Three studies focused on the archaeal lipidome and another explored an archaeal exometabolome. Two of these studies analyzed lipids extracted from ocean surface waters with abundant MGII *Euryarchaeota* populations. The first correlated surface planktonic communities dominated by MGII to a distinct lipidome dominated by acyclic isoprenoid glycerol dibiphytanyl glycerol tetraethers (GDGTs) with diglycosidic and monoglycosidic headgroups (Ma et al.). The second reported extremely low intact polar lipid content of GDGTs (1.21 × 10^−9^ ng lipid/cell) with surface planktonic communities from the North Pacific Subtropical Gyre comprised of *Thermoplasmatota* (MGII/MGIII) and devoid of AOA. This suggests a minimal contribution of *Thermoplasmatota* to GDGT pools in ocean waters, despite the detection of homologs of the archaeal GDGT ring synthase genes encoding GrsA and GrsB (Li et al.). A third paper reported on a novel application of liquid chromatography ion mobility mass spectrometry (IM-MS) to enhance the coverage and structural interpretation of archaeal lipids (Law et al.). The approach was then used to identify a novel phosphate- and sulfate-containing lipid in pure cultures of the AOA *Nitrosopumilus maritimus*. Finally, another paper characterized the exometabolome of *N. maritimus* by liquid chromatography coupled to IM-MS (Law et al.). The exometabolome was dominated by biologically active nitrogen-containing metabolites, peptides, cobalamin, and cobalamin biosynthetic intermediates, enforcing the important role of *N. maritimus* in oceanic carbon and nitrogen cycles and as a source of cobalamin. The latter two papers highlight the importance of developing new technologies in mass spectrometry for promoting research on archaeal lipidomics and metabolomics.

## Ecology of Archaea in Extreme and Terrestrial Environments

Archaea are traditionally viewed to be important members of microbial communities in extreme environments, such as terrestrial hot springs. This is still true, and the importance of archaea is expanded into other terrestrial environments, as exemplified by papers on archaea in acid mine drainage, shallow subsurface sediments, and suboxic swamp soils. One paper extends the AOA theme from oceans to terrestrial geothermal systems by describing nine new MAGs representing *Candidatus* Nitrosocaldaceae (Luo et al.). The genomes represent two novel species of *Candidatus* Nitrosocaldus and a member of a second genus, *Candidatus* Nitrosothermus. The genomes contained genes related to thermal adaptation and vitamin synthesis, including cobalamin, extending the possible role of AOA in cobalamin synthesis to thermophiles. A similar approach was used to obtain five MAGs from acid mine drainage sediments representing a novel family of the *Parvarchaoeta* (Luo et al.). Like other *Parvarchaeota* genomes, the new MAGs lacked amino acid biosynthetic pathways, but they did contain genes related to carbohydrate and protein utilization, adaptation to acid and heavy metals, and sulfocyanin, the latter suggesting energy conservation based on chemolithotrophic iron oxidation.

Two papers explored archaea in less extreme terrestrial environments. The first described archaeal and bacterial communities in oxic and anoxic clays from different depths in a terrestrial borehole in the Jianghan Plain, China (Song et al.). Archaea were abundant at all depths, comprising up to ~60% of the microbial community. The dominant community members were *Bathyarchaeota, Euryarchaeota, Thaumarchaeota*, and *Woesearchaeota*. Another paper quantified methanotrophy and nitrogen fixation and used stable isotope probing to identify active methylotrophs in suboxic alpine swamp soils of the Qinghai–Tibetan Plateau, *Methylobacter*-like bacteria as the dominant active population, rather than archaea (Mo et al.).

## Novel Metabolism and Nucleic Acid Biochemistry of Diverse Archaea

The Research Topic also included important contributions to archaeal physiology and nucleic acid biochemistry. One review article summarized mounting evidence for direct interspecies electron transfer (DIET) between methanogens and other microorganisms (Gao and Lu). The review article discusses several examples of DIET as an alternative to electron-transfer mediators like H_2_ or formate both for methanogenesis and AOM, and rare occurrences of membrane-bound multiheme c-type cytochromes (MHC) and electrically conductive cellular appendages that may mediate these processes in methanogens.

Two papers explored proteomic responses to stress in hyperthermophilic archaea from marine and terrestrial systems. The first examined multiple stress responses in the hyperthermophilic and piezophilic archaeon *Thermococcus eurythermalis* A501 (Zhao et al.). A high percentage of differentially abundant proteins were shared between thermal, hydrostatic, and salinity stresses, particularly those involved the biosynthesis and protection of macromolecules, amino acid metabolism, ion transport, and binding activities. Another paper probed the DNA damage response of *Sulfolobus islandicus* to UV-induced DNA damage by a quantitative phosphoproteomic analysis (Huang et al.). The study revealed 562 phosphorylated sites in 333 proteins, including 30 that were induced by UV treatment. Several of the UV-induced phosphorylations were dependent on an Rio1 kinase homolog, based on phosophorylation in the wild-type strain but not a deletion mutant of *rao1*.

Finally, three papers provided insights into nucleic acid biochemistry of archaea. The first shows one of the three B-family DNA polymerases of *S. islandicus*, Dpo2, to be the main DNA polymerase responsible for DNA damage tolerance (Feng et al.). Dpo2 expression was induced by DNA damage, enhanced viability to cells with DNA damage when overexpressed, and participated in mutagenic translesion DNA synthesis. Also in *S. islandicus*, Ye et al. used a combination of genetic and molecular biology approaches to show that the CRISPR-associated factor Csa3b is a repressor for acquisition of CRISPR spacers and a transcriptional activator of Cmr-mediated RNA interference, adding a key piece to the puzzle of the mechanism of immunity in *S. islandicus*. Finally, the characteristics of the archaeal RNaseZ in the maturation of 3'-ends of pre-tRNAs was investigated in *Methanolobus psychrophilus* and *Methanococcus maripaludis* (Wang et al.). Endoribonuclease activity was dependent on cobalt ions, independent of CCA motifs, and active on intron-embedded archaeal pre-tRNAs that are common in some archaea.

## Outlook on Archaeal Research and Global Cooperation

We are sure you will enjoy the diverse and exciting research contained in this Research Topic and share in our enthusiasm for continued international collaboration on all areas of archaeal biology. As exclaimed 4 years ago, “These are exciting times for archaeal research” (Adam et al., 2017). Continuing progress is expected to be made on archaea, particularly on archaeal evolution and systematics, the functions of major yet-uncultivated lineages, the role of archaea in eukaryogenesis, and lipidomics approaches as biomarkers for understanding ancient and modern archaeal communities.

In terms of evolution and systematics, we are seeing increased use of the Genome Taxonomy Database (GTDB) to aid in the taxonomic classification of both cultivated and uncultivated archaea (Rinke et al., 2021). The GTDB, along with the impending development of a code of nomenclature to name and catalog archaea and bacteria by using genome sequences as common currency (Murray et al., 2020), promises a more robust evolutionary framework and better communication among scientists studying archaea. We suggest that our research community may be early to adopt these systems compared to our counterparts studying bacteria due to the smaller size of the community and better appreciation for the prevalence of uncultivated archaea in nature and the power of meta-omics technologies to study them. These systems form a foundation for genome-focused studies across the domain, keeping in mind that some MAGs suffer from data quality problems not encountered in sequences from cultured organisms (Garg et al., 2020), particularly when closely related strains cohabitate and confound existing assembly and binning methods (Sczyrba et al., 2017). Problems related to MAG binning fidelity of short read assemblies are increasingly solved by including long-read sequencing data to produce hybrid assemblies (Frank et al., 2016; Xie et al., 2020; Ciuffreda et al., 2021), by utilizing Hi-C to assay the physical proximity of DNA sequences (DeMaere et al., 2020), and by employing single-cell genomics (Stepanauskas, 2012; Rinke et al., 2013; Pachiadaki et al., 2019).

Nevertheless, genomes from a plethora of both cultivated and uncultivated archaea discovered at an unprecedented rate (Nayfach et al., 2021; Rinke et al., 2021) are serving as invaluable resources for studying both extant organisms and for probing deep evolutionary relationships and key evolutionary transitions. Eukaryogenesis will continue to be a major research frontier in this area, but other important insights into archaeal evolution await. For example, recent progress on the genomic evolution of diverse archaea in the context of Earth history provide better ties between genome-based evolutionary models and the sedimentary record (e.g., Colman et al., 2018; Wang et al., 2021b; Yang et al., 2021). The fast-increasing volume and diversity of high-quality environmental genomes should lead to fruitful new ground in archaeal evolutionary studies along geological time scales, which has been hampered by the lack of physical fossils of microorganisms. We suggest that studies that integrate molecular and sedimentary records, such as lipid biomarkers, will be especially fruitful going forward.

Synthetic biology is also allowing breakthroughs in elucidating the long-standing enigma of the “lipid divide” that distinguishes the bacterial and archaeal cell membrane compositions (Martin and Russell, 2003; Villanueva et al., 2017; Caforio et al., 2018). These studies provide important insights into modern biology and improve interpretations of contemporary and fossil archaea and on the evolution of membrane biochemistry and function (Zeng et al., 2019). At the same time there is a need to distinguish between genomic potential for physiological traits and *bona fide* expressed traits *in situ* or in cultured cells. For example, Sun et al. (2021) and Villanueva et al. (2021) report exciting collections of genes from uncultured archaea that suggest the ability for coexistence of archaeal and bacterial lipids in the same cell. But do they really synthesize those lipids? In order to learn more, we need cultured cells.

As another example, He et al. (2016) reported the possibility of widespread acetogenesis in archaea based on genomic potential, but the only acetogenic growth of cultured archaea reported so far was obtained from laboratory cultures of a methanogen forced to grow on carbon monoxide (Rother and Metcalf, 2004). There is a clear gap between our ability to ascertain novel (meta)genomic potentials and our ability to link these inferred abilities to physiological traits in cultured cells. Two of the most prominent metagenomic-identified archaea from sediment that have been brought to culture so far are *Ca*. Korarcheaum cryptofilum (Elkins et al., 2008) and *Ca*. Prometheoarchaeum syntrophicum (Imachi et al., 2020), both of which grow from simple peptide fermentations, even though the former and its close relatives (McKay et al., 2019) possesses a number of genes for methanogenesis-related pathways (Xavier and Martin, 2019).

The flood of genomic data from archaea in new environments highlights the need to cultivate new archaea in both mixed and pure cultures in combination of traditional methods, and new genome-guided approaches (Wang et al., 2021a). The fore-mentioned *Ca*. Prometheoarchaeum syntrophicum can serve as a great example of successful cultivation, which grows very slowly to low cell density in culture with one or more hydrogenotrophs (Imachi et al., 2020). *Ca*. Prometheoarchaeum needed over 7 years of enrichment cultivation to gain sufficient purity and biomass for incisive experimentation. Despite being notoriously difficult to grow, *Ca*. Prometheoarchaeum syntrophicum and other challenging archaea (e.g., *Ca*. Korarchaeum) provide irreplaceable templates for physiological and biochemical studies of these novel organisms under controlled laboratory conditions. Thus, cultivation must be a priority for future research, and it will be a long-term endeavor. With all the exciting challenges in archaeal research, improved cooperation would be helpful to combine our expertise and accelerate archaeal research in the next decade. Borrowing a phrase from the early days of microbial ecology—“*Everything is everywhere*, but, *the environment selects*” (Baas-Becking, 1934)—archaea truly are everywhere and play important roles in any environment that supports them. Illuminating these roles can help scientists across the globe to identify and pursue common goals in archaeal research.

## Author Contributions

CZ initiated and organized the IWGOA and this Research Topic. BH wrote the first draft of the editorial article. CZ, FW, CR, and WM edited this editorial. All authors contributed to the article and approved the submitted version.

## Funding

The International Workshop on Geo-omics of Archaea was funded in part by the U.S. National Science Foundation (DEB 1928924), the National Science Foundation of China (No. 91851210), the Shenzhen Key Laboratory of Marine Archaea Geo-Omics, Southern University of Science and Technology (ZDSYS201802081843490), and the Southern Marine Science and Engineering Guangdong Laboratory (Guangzhou) (No. K19313901).

## Conflict of Interest

The authors declare that the research was conducted in the absence of any commercial or financial relationships that could be construed as a potential conflict of interest.

## Publisher's Note

All claims expressed in this article are solely those of the authors and do not necessarily represent those of their affiliated organizations, or those of the publisher, the editors and the reviewers. Any product that may be evaluated in this article, or claim that may be made by its manufacturer, is not guaranteed or endorsed by the publisher.
